# Longitudinal Evaluation of the Hypothalamic-Pituitary-Testicular Function in 8 Boys with Adrenal Hypoplasia Congenita (AHC) Due to *NR0B1* Mutations

**DOI:** 10.1371/journal.pone.0039828

**Published:** 2012-06-27

**Authors:** Caroline Galeotti, Zineb Lahlou, Domitille Goullon, Hélène Sarda-Thibault, Juliette Cahen-Varsaux, Joëlle Bignon-Topalovic, Anu Bashamboo, Ken McElreavey, Raja Brauner

**Affiliations:** 1 Université Paris Descartes, Sorbonne Paris Cité, and Assistance Publique-Hôpitaux de Paris, Hôpital Bicêtre, Unité d’Endocrinologie Pédiatrique, Le Kremlin Bicêtre, France; 2 Centre hospitalier René Dubos, Service de Pédiatrie, Pontoise, France; 3 Centre hospitalier Victor Dupuy, Service de Diabétologie-Endocrinologie, Argenteuil, France; 4 Human Developmental Genetics Unit, Institut Pasteur, Paris, France; Baylor College of Medicine, United States of America

## Abstract

**Background:**

Boys carrying mutations in the *NR0B1* gene develop adrenal hypoplasia congenita (AHC) and impaired sexual development due to the combination of hypogonadotropic hypogonadism (HH) and primary defects in spermatogenesis.

**Methods:**

We analysed the evolution of hypothalamic-pituitary-testicular function of 8 boys with AHC due to *NR0B1* mutations. Our objective was to characterize and monitor the progressive deterioration of this function.

**Results:**

The first symptoms appeared in the neonatal period (n = 5) or between 6 months and 8.7 years (n = 3). Basal plasma adrenocorticotrophic hormone (ACTH) concentrations increased in all boys, whilst cortisol levels decreased in one case. The natremia was equal or below 134 mmol/L and kaliemia was over 5 mmol/L. All had increased plasma renin. In 3 of 4 patients diagnosed in the neonatal period and evaluated during the first year, the basal plasma gonadotropins concentrations, and their response to gonadotropin releasing hormone (GnRH) test (n = 2), and those of testosterone were normal. The plasma inhibin B levels were normal in the first year of life. With the exception of two cases these concentrations decreased to below the normal for age. Anti-Müllerian hormone concentrations were normal for age in all except one case, which had low concentrations before the initiation of testosterone treatment. In 3 of the 8 cases the gene was deleted and the remaining 5 cases carried frameshift mutations that are predicted to introduce a downstream nonsense mutation resulting in a truncated protein.

**Conclusions:**

The decreases in testosterone and inhibin B levels indicated a progressive loss of testicular function in boys carrying *NR0B1* mutations. These non-invasive examinations can help to estimate the age of the testicular degradation and cryopreservation of semen may be considered in these cases as investigational procedure with the aim of restoring fertility.

## Introduction

The DAX-1 (dosage sensitive sex reversal, adrenal hypoplasia congenita, critical region of the human chromosome, gene 1) protein is an orphan member of the nuclear receptor superfamily. It is encoded by the *NR0B1* gene, which is expressed in the adrenal cortex, gonads, hypothalamus and anterior pituitary [1]. Boys carrying mutations in the *NR0B1* gene develop adrenal failure (adrenal hypoplasia congenital, AHC) and showed impaired sexual development at puberty, followed by infertility. This impairment is due to the combination of hypogonadotropic hypogonadism (HH) [2], [3], [4] and a primary defect in spermatogenesis [5]. Male mice lacking Dax-1 are infertile due at least partly to blockage of the rete testis, with subsequent dilatation of the proximal efferent ductules [6]. To date, more than 100 different mutations in *NR0B1* have been described [7] and it has been estimated that 58% of boys with idiopathic primary adrenal insufficiency have mutations in *NR0B1* [8].

In infants with AHC associated with *NR0B1* mutations the plasma basal testosterone, luteinising hormone (LH) and follicle stimulating hormone (FSH) concentrations are within the normal range and these increase following the gonadotropin releasing hormone (GnRH) test [9], [10], [11]. Immunohistochemical analysis of testicular tissue obtained from an affected sibling of one boy with an *NR0B1* mutation, who died with adrenal failure as a neonate, showed normal testicular morphology with numerous Sertoli cells, and expression of DAX-1, steroidogenic factor-1 (SF-1), and anti-Müllerian hormone (AMH) proteins [12]. Conversely, in a 20- yr old man, a testicular biopsy revealed disorganization of the normal seminiferous tubular structure and moderate Leydig cell hyperplasia [13] similiar to the phenotype described in the *Nr0b1* knockout mouse model [5], [6]. Recently, Frapsauce et al [14] reported birth after testicular sperm extraction (TESE)-ICSI from a 25- yr old azoospermic man with HH and AHC due to an *NR0B1* mutation.

It is difficult to predict the reproductive capacity of a child before pubertal age because the plasma concentrations of FSH and LH are not informative and semen analyses cannot be done. The plasma concentrations of inhibin B and AMH might be helpful at this age. In boys, inhibin B is produced by the Sertoli cells. Its plasma concentration is the best plasma marker of spermatogenesis [15], [16], [17]. Individual data on the plasma inhibin B and AMH concentrations in patients with *NR0B1* mutations have been reported [13], [18], [19], but not during infancy and childhood.

We analysed the evolution of hypothalamic-pituitary-testicular function from the diagnosis to young adulthood of 8 boys with AHC due to *NR0B1* mutations, by assessing their plasma inhibin B and AMH concentrations. Our objective was to characterize and monitor the progressive deterioration of this function.

## Results

### Clinical and Biological Presentation (Table 1)

**Table 1:** Clinical and biological data of 8 boys with DAX-1 mutations.

Cases	1	2	3	4	5	6	7	8
**Origin**	France	Morocco	Morocco	France	Martinique/Guadeloupe	Martinique/Guadeloupe	France/India	Spain/Guinea
**Age of onset**	neonate	neonate	neonate	neonate	neonate	6 mo	1.3 yr	8.7 yr
**Age of diagnosis**	neonate	neonate	neonate	neonate	neonate	3.5 yr	1.5 yr	8.7 yr
**First symptoms**	vomiting	vomiting,melanodermia	systematic screening	vomiting, hypothermia	vomiting	vomiting, seizures	vomiting, seizures	vomiting, abdominal pain, seizures, melanodermia
**Glucose, mmol/L**	3.6	3.3	2.3	NA	NA	2.8	3.5	2.9
**ACTH, pg/mL**	242	1500	1850	120	980	4000	11649	405
**Cortisol, ng/mL**	100	43	45	28	166	98	99	225
**Sodium, mmol/L**	129	129	138	116	121	119	119	134
**Potassium, mmol/L**	7	5.5	4.3	9.6	6.8	5.7	5.2	5.8
**Renin, pg/mL**	90	480	173.5	NA	NA	NA	NA	675
**Plasma renin activity, ng/mL/h**	NA	NA	NA	>100	70	48	23	NA
**Aldosterone, pg/mL**	NA	7.5	NA	30	74	NA	36	42
**Age of last evaluation, yr**	18.8	7.1	2	17.3	18.8	21.3	16.4	29.7
**Adult height, cm**	169.5			173	167.5	177.5		177
**Target height, cm**	175	178.5	178.5	181.5	180	NA	174	177.5

Normal Range: Renin, 3–16 pg/mL; Plasma Renin Activity, 0.2–2.80 ng/mL/h and 0.8–8.9 ng/mL/h during the first year of life; Aldosterone: 42–201 pg/mL.

Case 1 has maternal uncle with DAX1 mutation and azoospermia. Case 7 has maternal cousin with DAX1 mutation.

Cases 2 and 3, and 5 and 6 are brothers.

Intra-uterin growth retardation in cases 4 and 7.

Six out of the 8 patients had a familial history of AHC. The pregancy, delivery and mensurations at birth were normal, except in cases 4 and 7 whose height and weight at birth were at –2 standard deviations.

The first symptoms appeared in the neonatal period (5 patients) or between 6 mo and 8.7 yr (3 patients). In these, the diagnosis was made between 1.5 and 8.7 yr. In case 3, the diagnosis was made at birth by systematic screening after the brother’s history. In the 7 others, the presenting symptom was vomiting, associated with hypothermia (one case), abdominal pain (one case) and/or seizures (three cases).

The plasma glucose concentration was equal or below 3.6 mmol/L in the 6 cases that were evaluated. Basal plasma ACTH concentrations were increased in all boys, while that of cortisol was decreased only in case 4. The natremia was equal or below 134 mmol/L and kaliemia was over 5 mmol/L in all boys except in case 3, who was diagnosed at birth by systematic screening. All boys had increased plasma renin or renin activity. The blood creatin phosphokinase enzyme and urine glycerol were normal in all cases, indicating that there was no contiguous deletion syndrome with Duchenne muscular dystrophy or glycerol kinase deficiency. Five patients had an abdominal computed tomography scan or an echography, which revealed small adrenal glands in one patient (case 2), normal adrenal glands in 2 patients (cases 4 and 7) and the adrenal glands were not seen in 2 patients (case 1 and 5).

Treatment with Hydrocortisone® and 9-alpha fludrocortisone was started in emergency in all patients. The Hydrocortisone® replacement dose was adjusted on the basis of plasma ACTH concentrations and that of 9-alpha fludrocortisone on renin or renin activity. Careful and repeated information was given to the parents and patients on the need to increase the dose of Hydrocortisone® in case of specific events, and to replace both Hydrocortisone® and 9-alpha fludrocortisone with injections if there were any gastrointestinal symptoms or anaesthesia. The psychomotor development of all the patients was normal. Five patients reached their adult height, which was lower than their target height, except for the case 8.

### Hypothalamic-pituitary-testicular Function (Table 2)

**Table 2:** Hypothalamic-pituitary-testicular function of 8 boys with DAX-1 mutations.

Cases	1	2	3	4	5	6	7	8	
**Testicular volume, mL**	12.8 yr:4.5			10 yr:213.9 yr:415.7 yr:5	11.6 yr: 4	13.6 yr:6 14.3 yr:6	15 yr:3.8 16.4 yr:5.4	15.7 yr:2 17.8 yr:3	
**FSH basal/peak, IU/L**	2 mo:2.6/3.9 14.8 yr:<0.4/<0.4 18.8 yr:1.3/1.7	6 mo:1.9/3.3	8 d: 1.2	13 d:2 13.9 yr:3/4 16.8 yr:5 19.8 yr:1	NA	14.3 yr:2.3/3.3	13.7 yr:0.9/1.616.4 yr:1.6	15.7 yr:1.19/1.62	
**LH basal/peak, IU/L**	2 mo:.1/614.8 yr:<0.4/<0.418.8 yr:0.1/0.3	6 mo:1.2/4.5	NA	13 d:1.1 13.9 yr:1/1 16.8 yr:4 19.8 yr:<1	NA	14.3 yr:0.78/1.7	13.7 yr:<0.4/<0.4 16.4 yr:0.6	15.7 yr:0.4/0.67	
**Testosterone, ng/mL**	2 mo:3.714.8 yr:<0.05	29 d:1.8 6 mo:<0.05 7.1 yr:<0.02	8 d: 1.1	13 d:1.8 10 yr:<0.04 13.9 yr:0.6 15.7 yr:3 16.8 yr:4.1 17.3 yr:0.1	14 d:0.1613.4 yr:0.0513.8 yr:0.16	13.6 yr:0.114.3 yr:0.6714.7 yr:<0.07	9.7 yr:<0.0515 yr:<0.0216.4 yr:0.02	13 yr:<0.05 14.3 yr:<0.05 15.7 yr:<0.04	
**Inhibin B, pg/mL**	17 d:259 14.8 yr:103	9 mo:250 7.5 yr:19	8 d:225 6 mo:343 2.3 yr:27	NA	11.6 yr:6513.8 yr:20	16 yr:28	9.7 yr:121 13.7 yr:95 16.4 yr:94	29.7 yr:20	
**AMH, pmol/L**	17 d:700 14.8 yr:103	9 mo:1015 7.5 yr:252	8 d:264 6 mo:740 2.3 yr:284	NA	11.6 yr:27	NA	9.7 yr:213 13.7 yr:237	20 yr:41029.7 yr:410	
**Age at onset of testosterone, yr**	14.8			17.3	13.8	14.7	15	15.8	
**DAX mutation**	?	2 µdel	2 µdel	Complete deletion	µdel	µdel	Del 651	Insertion 284	

Normal range: Inhibin B, pg/mL, 12 mo, 94–383, 24 mo, 71–204, 5–10 yr, <20–258, adult, 80–270; AMH, pmol/L, 10d-1 yr, 390–570, 1 yr–4 yr, 360–650, 4 yr–7 yr, 320–560, 7 yr–9 yr, 240–430, adult, 22–38.

Unilateral cryptorchidism in cases 2 and 3.

Two brothers (cases 2 and 3) had unilateral cryptorchidism and one of them (case 3) had been operated on in emergency at two years of age for torsion of the inguinal testis. The volume of their other, intrascrotal testis was 2 mL. The size of the penis was normal in all of the boys. Cases 5 and 7 had pubic hair development at 11 and 7 yr, despite the absence of any familial history of early puberty.

In the 4 patients diagnosed in the neonatal period and evaluated during the first year, the basal FSH and LH, and their response to the GnRH test (n = 2), and the basal plasma testosterone concentrations were normal, except for case 5 who had testosterone at 0.16 ng/mL at 14 days. With one exception, the testicular volumes did not increase at pubertal age and the plasma concentrations of FSH, LH and testosterone were very low or undetectable, including during the early puberty in case 7. Case 4 had clinically and biologically onset of puberty at 13.9 yr, followed by clinical arrest. The plasma concentrations of FSH, LH and testosterone decreased to undetectable levels, leading to testosterone substitution at 17.3 yr.

The plasma inhibin B concentrations were within the normal range in the first year of life, with a normal increase at 6 months in case 3 (Table 2). These concentrations decreased to below the normal range for age in all cases, except for the cases cases 1 and 7. The plasma AMH concentrations were normal for age in all individuals except case 5 who had low concentrations before the initiation of the testosterone treatment.

### Genetic Analyses

All 8 cases carried severe mutations in the *NR0B1* gene.

**Figure 1:**
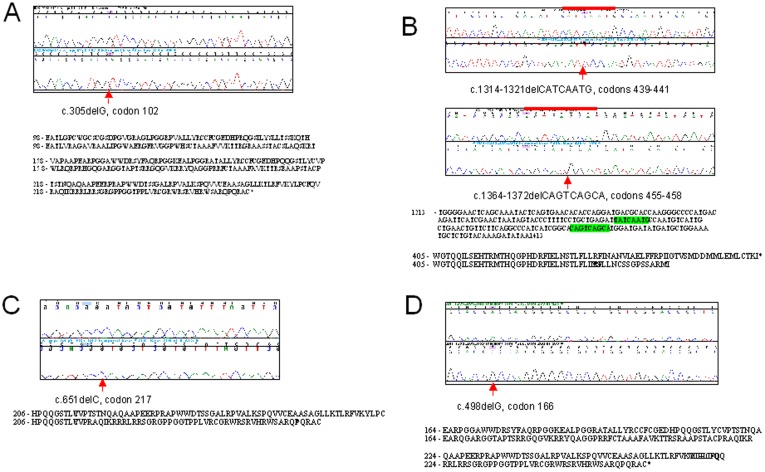
Mutations in DAX1 associated with AHC. Panels A, B, C and D correspond to patients 1, 3/4, 7 and 8 in table 1 respectively. For each mutation a representative chromatogram is shown together with the sequence of a male control. The position of the mutation is indicated by a red arrow and/or line. The consequences of the frameshift mutations on the predicted amino acid sequence is shown under each chromatogram. In panel B, the deletion in the cDNA sequence is indicated by the highlighted bases.

Case 1 is hemizygote for a c.305delG mutation at codon 102. This mutation is predicted to result in the generation of a downstream stop codon at position 263 (Fig 1a).

Cases 2 and 3 are brother carrying two hemizygous mutations in exon 2. The first is an 8 bp deletion, c.1314–1321delCATCAATG at codon positions 439–441. The second mutation is a 9 bp deletion, c.1364–1372delCAGTCAGCA at codons 455–458. These two frameshift mutations are predicted to alter the protein sequence and result in a premature stop codon at position 455. The mother was found to be heterozygous for both of these deletions (Fig 1b).

Case 4, 5 and 6 have complete deletions of the *NR0B1* gene. Case 5 (brother of case 6) have been previously reported [3] and the mother was found to be heterozygous for these deletions.

Case 7 carries a hemizygous c.651delC mutation at codon 217. Again this deletion is predicted to alter the protein sequence and generate a premature stop codon at position 263 (Fig 1c).

Case 8 is hemizygous for a c.498delG mutation at codon position 166. This mutations is predicted to generate a stop codon at amino acid position 263 (Fig 1d).

In all of the boys the *NR5A1* gene was sequenced as described elsewhere [25]. No mutations were observed in the gene.

## Discussion

This study shows that the plasma inhibin B, testosterone, LH, FSH and AMH concentrations are normal in the first year of life, and interestingly there is a normal increase in inhibin B concentration that occurred at 6 months. The two patients evaluated by GnRH test at this age had a normal response with the LH peak greater than the FSH peak. A decrease of these concentrations occurred in all the boys, with no pubertal increase of testosterone, except a transient normal increase in one, leading to delayed substitutive testosterone treatment. The AMH concentrations remained normal and did not decrease despite the testosterone treatment. Only two patients maintained normal plasma inhibin B concentrations at 14.8 and 16.4 yr. No spermogram was performed, but the maternal uncle of one of these with normal inhibin B, sharing the same *NR0B1* mutation, is azoospermic.

### Clinical and Biological Presentation

The age at the first symptom in two brothers was neonate and 6 months. The intrafamilial variation of the age at presentation had been reported in many unrelated families with different mutations [10], [26]. The presenting symptom was vomiting in all cases, associated with seizures in three. All 6 evaluated had hypoglycaemia. The seizures are probably due to the combination of the hypoglycaemia and hyponatremia. Hypoglycemia is suggestive of cortisol deficiency, and may be associated in the neonates with jaundice and/or low blood pressure, or it may be isolated as observed in the present cases. Profound persistent hypoglycemia is a clear sign if it is not explained by other neonatal conditions. As hypoglycemia is the best stimulus of ACTH and cortisol secretion, their plasma concentrations should be measured during the spontaneous hypoglycaemia whatever is the age of the patient.

The basal plasma ACTH concentrations are greatly increased in our patients, and in those reported elsewhere. These high concentrations stimulate cortisol secretion, explaining the normal plasma cortisol concentrations in most of our patients, but these nomal concentrations are insufficient to maintain normal glucose concentrations and to prevent the seizures. In addition, the patients are exposed to acute adrenal insufficiency [20].

### Hypothalamic-pituitary-testicular Function

In a normal infant, the plasma testosterone concentration increases to the adult level at birth by stimulation of the Leydig cells by human chorionic gonadotropin originating from the placenta. This is followed by a transient postnatal activation of the hypothalamic-pituitary-testicular axis. Thus, there is a bimodal pattern in testosterone in male infants during the first year of life: from birth to about the second month, a gradual increase in testosterone occurs [24]. From the first to third months, the mean testosterone level is significantly higher than at two weeks of life. After one to three months, there is a gradual decrease until the seventh month. From 7 to 12 months, the plasma testosterone concentrations are similar to those of prepubertal children. The increases in testosterone concentrations are associated with LH and FSH concentrations significantly higher than in the prepubertal age.

In the present study, 4/5 cases had normal testosterone concentration during the first months, as were the basal and GnRH stimulated LH and FSH levels. This was followed by a decrease with secondary deficit. Only one case, with a deletion of the *NR0B1* gene, had a spontaneous puberty with a slight increase in testicular volume, followed by a decrease of basal concentrations with decrease in testosterone from 4.1 ng/ml at 16.8 years to 0.1 ng/ml at 17.3 years. Binder et al [27] also reported transient onset of clinical and biological puberty in boys with *NR0B1* mutations.

Habiby et al [28] have shown the phenotypic heterogeneity of AHC/HH, and suggest that *DAX-1* mutations impair gonadotropin production by acting at both the hypothalamic and pituitary levels. They showed that administration of human chorionic gonadotropin (3 x 2,000 U each day) increased the plasma testosterone concentration of their patient from 0.4 to 6.79 ng/ml by day four.

In the literature, two brothers were reported with cryptorchidism and two patients had early pubertal development followed by gonadotropins deficiency [11], [26], [28]. Tabarin et al [19] reported a man who had a AHC with an *NR0B1* mutation and with a delayed-onset primary insufficiency in adulthood and an incomplete HH. His inhibin B was dramatically decreased at 7 pg/ml. They suggested that *NR0B1* should be considered a candidate gene in young adults presenting with mild “idiopathic” HH.

### Degradation of the Testicular Function

We evaluated the Sertoli cell function by dosing inhibin B and AMH in all but one patient. The plasma inhibin B concentrations were normal in the first year of life, with normal increase at 6 months in one case. These concentrations decreased to below the normal for age in all, except in two cases. Those of AMH were normal for age in all except one case who had low concentrations before the initiation of the testosterone treatment. As plasma inhibin B levels reflect the spermatogenic status throughout the sexual maturation [29], [30], our longitudinal data suggest that spermatogenesis is normal at least during the first year of life, followed by a degradation.

The testis of the Dax-1-deficient mice were initially shown to be small with degeneration of the seminiferous epithelium and loss of germ cells [6]. Jeffs et al [6] showed that there were poorly differentiated Sertoli cells within the lumen of the seminiferous tubules and efferent ducts and Leydig cell hyperplasia. Meeks et al [31] have demonstrated that DAX-1 plays a crucial role in testis differentiation by regulating the development of peritubular myoid cells and the formation of intact testis cords. Indeed, in this study, *NR0B1* deficiency was associated with defective development of testis cords and a decreased number of peritubular myoid cells.

In the Dax-1-deficient mouse, gonadal development is apparently normal until the point of testis cord formation, suggesting that DAX-1 plays a role in gonadal differentiation rather that gonadal patterning or sex determination. *NR0B1* mutations in humans result in HH and infertility rather than disorder in sex differentiation.

Mantovani et al [32] have shown that there is a gonadal dysgenesis that is independent of the gonadotropin deficiency. Indeed, they have treated a 28 years old man for hypogonadism due to an *NR0B1* mutation with gonadotropins during 8 months and he did not achieve fertility after this treatment. Treatment with exogenous gonadotropins stimulates testosterone production but does not appear to normalize spermatogenesis in the few individuals who have been carefully studied.

## Materials and Methods

### Ethics Statement

Written informed consent for the evaluation and molecular analyses was obtained from the parents. All clinical investigations were conducted according to the principals expressed in the Declaration of Helsinki. The Ethical Review Committee (Comité de Protection des Personnes, Ile de France III) approved the study. This stated that “this research was found to conform to generally accepted scientific principles and research ethical standards and to be in conformity with the laws and regulations of the country in which the research experiment was performed”.

### Patients

This single-centre retrospective case-cohort study was carried out on eight consecutive patients with AHC due to *NR0B1* mutations seen by a senior pediatric endocrinologist (R Brauner) from 1990 to 2011. We excluded all patients presenting with secondary adrenal failure due to adrenocorticotrophic hormone (ACTH) deficiency (n = 8), or primary adrenal failure associated with gonadal dysgenesis and disorder of sexual development (n = 5 from 4 families), adrenoleukodystrophy (augmentation of very-long-chain fatty acids, n = 3), autoimmune Addison’s disease (association with other autoimmune manifestations, n = 5), IMAGE syndrome (n = 1), or congenital adrenal hyperplasia (increased concentrations of 17 hydroxyprogesterone, 11-desoxycortisol, 11-desoxycorticosterone or dehydroepiandrosterone). The etiology in three other patients seen during the same period with primary adrenal failure who did not carry *NR0B1* mutations remains undetermined.

### Methods

Diagnosis of primary adrenal failure was based on increased plasma basal ACTH concentrations (normal 10–50 pg/mL). The concomitant cortisol concentrations were considered as low when below 40 ng/mL in the newborn or below 80 ng/mL in the older children in spontaneous hypoglycemia and/or at 8.00 a.m. Diagnosis of mineraloid deficiency was based on relatively low aldosterone concentrations in face of increased renin or plasma renin activity, with or without hyponatremia and/or hyperkaliemia [20]. Pubertal development was considered as early when pubic hair development began before 11 years.

All boys, except the cases 2 and 3, reached the pubertal age. They were given testosterone enanthate (25 mg i.m. every 14 days) after 13 years of age for low gonadotropins and testosterone. The doses were increased to adult levels (250 mg i.m. every 21 days) after the end of their growth. All plasma samples were collected before or at least one month after testosterone administration in the patients given testosterone.

Plasma hormone concentrations were measured using different immunoassays during the study period. A new assay method for a given hormone was always cross-correlated with the previous method. Thus, the results are comparable throughout the whole period. Basal plasma concentrations of FSH, LH and testosterone were measured in the first months of life, in the patients with early pubertal development and/or at the pubertal age. At least one GnRH test was performed in 6 patients. An aliquot of plasma was frozen at −20°C to measure inhibin B [21] and AMH [22]. In the first month of life, we used [23] and the first year [24] as normal values.

Inhibin B was measured in the serum by an enzyme immunometric assay (Oxford Bio-Innovation reagents, Serotec, Oxford, UK). AMH was measured by an enzyme immunometric assay (Immunotech reagents; Beckman Coulter Company, Marseille, France). FSH and LH were measured with a sensitive immunoradiometric assay (CIS bio international, GIF sur Yvette, France), with a detection limit of 0.05 IU/L in both assays (IU/L 2^nd^ IRP WHO 78/549 for FSH, IU/L 1 ^st^ IRP 68/40 for LH).

### Sequence Analyses

DNA was isolated from peripheral blood lymphocytes using standard techniques. The sequence analysis of the NR5A1 gene was performed as described elsewhere [25]. The following primers were used to amplify the *NR0B1* gene: Exon1F1 5′-TAGAAAGTCTGGTTGGAGCCTGAGATTG-3′. Exon1R1 5′-GCGTTTGCTTTGAGCTAGTGAGCAAG-3′ (1033 bp). Exon1F2 5′-GCTTTTGCGGTAAAGACCACCCAC-3′, Exon1R2 5′-TCCTGATCACTGAAAACTCGTTACTGCC-3′ (1071 bp). Exon2F1 5′-TTGCTTCTGAAAACGTAGACTGGTTTGG-3′, Exon2R25′-ACAGAGCTATGCTACCTGTTGGCAAATG-3′ (628 bp).

The PCR amplification conditions were, −95°C for 5 min followed by 37 cycles of 98°C 10s, 68°C 20 s, 72°C 1 min. A final elongation step of 72°C for 10 min was added in each case. 5 µl of each PCR fragments were then migrated in a 2% agarose gel stained in ethidium bromide (1 µg/mL) to verify the expected length of the amplified fragments. DNA sequence analysis was performed using at least 200 ng of purified DNA, 20 ng of primer and fluorescently labelled Taq DyeDeoxy terminator reaction mix (Applied Biosystems, Foster City, CA, USA) according to the manufacturer’s instructions. DNA sequence was determined using an ABI 3700 automated DNA sequencer.

### Conclusion

The decreases in testosterone and inhibin B levels indicated a progressive loss of testicular function in boys carrying *NR0B1* mutations. The limitation of this retrospective study is that measurements are not standardised in timing. These examinations are interesting because they are non-invasive, and if they are systematically made, they can be used to estimate the age of the testicular degradation and plan for the cryopreservation of semen as an investigational procedure with the aim of restoring fertility in this group.
